# The Treatment of Hemorrhoids

**Published:** 1907-03

**Authors:** 


### The Treatment of Hemorrhoids.

M. R. DINKELSPIEL, in a paper on "The Medicinal Treatment of Hemorrhoids without Surgical Intervention" (*Therapeutic Medi-*

*cine,* January 1907), says that he regards non-operative treatment indicated in incipient forms, when the hemorrhoids are secondary to other disease, as hepatic cirrhosis, tumors, and the like, and when the hemorrhoids occur in aged individuals who cannot safely undergo operation. Constipation is a most potent cause, and it must be cured; the defecations being so arranged that they occur at night, as the subsequent rest relieves engorgement.

Locally, cleanliness is of primary importance; the parts should be washed with witchhazel solution, of which 1 or 2 ounces may also be injected into the rectum. Of late he uses the bismuth iodoresorcinsulphonate suppositories (anusol), which relieve the congestion and inflammation and liquefy the feces. When there also exists external inflammation, he slightly warms a suppository and gently annoints the parts. Under this treatment he has seeen many cases recover without recurrence.

---

TREATMENT OF ALCOHOLIC HEPATITY.

Dr. Fred C. Thum, Louisville, writes on "Subacute Alcoholic Hepatitis" (*Medical Progress,* February 1907) and describes it as a form intermediate between the acute hepatitis from a single protracted spree and chronic hepatitis from long years of alcoholic excess. Calomel, podophyllin, and similar drugs are not of very great use. Strikingly effective are pills consisting of salicylic acid, acid sodium oleate, phenolphthalein and menthol (probilin). They increase biliary secretion and render the bile sterile, thus inhibiting the inflammation in the hepatic structures.

